# A glitch in the matrix: Age-dependent changes in the extracellular matrix facilitate common sites of metastasis

**DOI:** 10.1002/aac2.12013

**Published:** 2020-10-07

**Authors:** Gloria E. Marino, Ashani T. Weeraratna

**Affiliations:** 1 Department of Biochemistry and Molecular Biology, Johns Hopkins Bloomberg School of Public Health, Baltimore, Maryland; 2 Department of Oncology, Sidney Kimmel Cancer Center, Johns Hopkins School of Medicine, Baltimore, Maryland

**Keywords:** aging, cancer biology, metastasis, microenvironment

## Abstract

People over 55 years old represent the majority of cancer patients and suffer from increased metastatic burden compared to the younger patient population. As the aging population increases globally, it is prudent to understand how the intrinsic aging process contributes to cancer progression. As we age, we incur aberrant changes in the extracellular matrix (ECM) of our organs, which contribute to numerous pathologies, including cancer. Notably, the lung, liver, and bone represent the most common sites of distal metastasis for all cancer types. In this review, we describe how age-dependent changes in the ECM of these organs influence cancer progression. Further, we outline how these alterations prime the premetastatic niche and why these may help explain the disparity in outcome for older cancer patients.

## Graphical Abstract

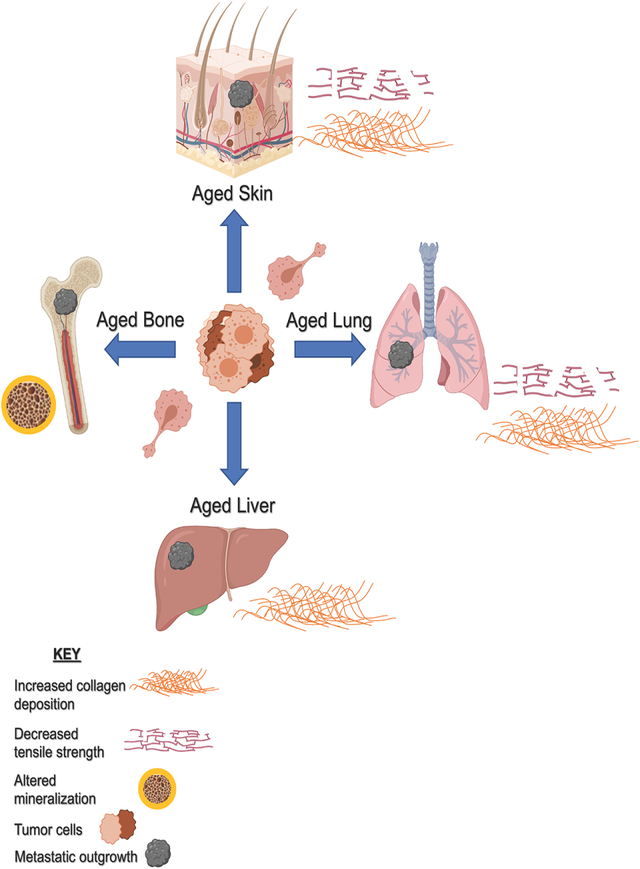

## 1 | INTRODUCTION

Aging is the inevitable progressive decline in intrinsic physiological function over time, leading to an increase in pathologies, including cancer. The incidence and probability of developing most cancers increases with age^1^ and over 78% of all cancers are diagnosed in people 55 years and older.^2^ Of those who die of cancer worldwide, 88% are over the age of 50.^3^ Importantly, the aging population is growing globally. By 2050, nearly 1.5 billion people are expected to be over 65 years old, which represents a 300% increase from 2010.^4^ This highlights the looming public health crisis of cancer care and management in the elderly.

Despite recent advances in the survival rates for most cancers diagnosed at early stages, metastasis is still responsible for the majority of cancer deaths.^5^ Although many of the mutations that cause cancer are due to random chance,^6^ the patterns of subsequent cancer dissemination are decidedly nonrandom. In 1889, Paget coined the famous “seed and soil” hypothesis, suggesting that cancer cell “seeds” are widely spread throughout the body as tumors grow, but they can only live and grow in appropriate organ “soil” sites.^7^ Apart from the lymph nodes, the three most common sites of metastasis for all cancer types are the lung, liver, and bone, which together make up nearly 30% of all distal metastases.^8,9^ Thus, these organs seem to have extraordinarily fertile soil independent of the innate properties of the seeds that encounter it. This begs the question, what makes these sites—this soil—different?

A critical component of what makes up our organ systems is the extracellular matrix (ECM), the three-dimensional network of macromolecules that provide structural and biomechanical support to the organs they serve. In essence, if organs are the soil then the ECM is what makes the soil healthy—the detritus, moisture level, and nutrients that all support overall soil fertility. In fact, the idea that the ECM is the basis of all life predates cell theory by nearly 150 years.^10^ It has long been recognized then that connective tissue is indispensable for providing a platform from which living things can arise.

The ECM shows incredible organ specificity throughout the body. A complex interplay between cells and the microenvironment produces ECM with distinct components, molecular distribution, and topography that are poised to perfectly support the biomechanical function of the organ.^11,12^ In a landmark paper, Hanahan and Weinberg posited “the hallmarks of cancer,” which enumerated six capabilities acquired by all cancer cells.^13^ It has been shown that both aging and the ECM provide biophysical and biochemical cues to influence and potentiate each of these hallmarks, which we discuss in further detail below.^14,15^ As we undergo the aging process, there are corresponding intrinsic changes in the ECM throughout the body that inhibit the biomechanical function of these organ systems.^16^ Notably, dysregulation of ECM components leads to aberrant interactions and mechanical deficiency, which puts these organs at higher risk for pathogenesis and more susceptible to cancer cell colonization and metastatic outgrowth. Thus, the intersection of aging and the ECM, and how these each influence one another, is critical for our understanding of malignancy and metastasis.

This review will focus on how age-dependent changes in the ECM of the lung, liver, and bone uniquely position these sites to be susceptible to cancer metastasis. Specifically, we will focus on how the age-dependent aberrancies in the ECMs of these tissues are critical in forming the premetastatic niche, and perfectly prime them to become sites of metastatic outgrowth.

## 2 | ECM AND AGING

ECM is the noncellular component of tissues and organs that provides a scaffold for tissues as well as critical cellular cues that support biological function.^17^ Notably, the composition, biomechanics, and supramolecular structure of the ECM are specific to support the function of each tissue. Generally, the components of the ECM are composed of two classes: fibrous proteins and proteoglycans (or nonfiber forming proteins).^18^ Due to the insoluble nature of many ECM components, it is challenging to form a complete component list for all organ-specific ECMs. Generally speaking, the core components required for any ECM are collagens, proteoglycans, glycoproteins, growth factors, and ECM modifiers.^12,19^ However, over 1000 ECM proteins have been identified within “the matrisome” and are catalogued at http://matrisomedb.pepchem.org/ where one can search by species, tissue, and disease.^20^ Among all organ-specific ECMs, the ECM of the dermis has been the most extensively described due to ease of organ collection, especially in the context of molecular and cellular aging. These changes over time encompass the accumulation of mutations over many years, including changes in secretory functions as well as the loss or gain of molecules involved in both signaling and biophysical cues. The loss or gain of ECM molecules in particular can directly impact tumor cells through altered signaling. For example, in the case of the skin ECM, young skin presents a highly organized matrix that is rich in collagen and aged skin presents a degraded ECM with fewer fibrillar components and less structural integrity.^21^ Our laboratory has found that HAPLN1, a proteoglycan linker protein, is lost during aging and renders the ECM less tightly organized with lower integrity.^21^ This affects not only the way tumor cells move in this ECM, but also the integrity of lymphatic vessels and immune cell motility.^21,22^ Aged skin is thus characterized primarily by alterations in the dermal ECM that lead to aberrancies in skin biomechanics such as increased stiffness and decreased elasticity.^23^ This is largely due to alterations in the secretomes of dermal fibroblasts, which produce the ECM. Aged fibroblasts decrease production of both ECM components, such as collagen, as well as ECM modifiers.^21,24,25^ The resulting degradation of the ECM is known to be a critical determinant for progression of the skin cancer, melanoma. In addition to altered secretomes, dermal fibroblasts become less dense with age and have disrupted homeostasis.^26^ These sorts of changes, in addition to the accumulation of chronic genetic damage, changes in adaptive immunity, and other insults, may explain why aging has long been considered an independent prognostic factor in melanoma whereby older patients have poorer prognoses.^27–29^

The complex and dynamic meshwork produced by the matrisome provides mechanical integrity and cellular regulation at an organ-specific level.^15^ This concept has been dubbed “dynamic reciprocity,” or where ECM/cell crosstalk potentiates function. The ECM exerts physical and chemical influence on the cell, which in turn alters the cell’s interaction with the ECM.^30^ This give-and-take phenomenon is also referred to as “mechanotransduction,” which is necessary for the proper biomechanical function of organs and tissues to support whole-body health.^31^ However, as we age, these complex networks begin to break down. This breakdown largely occurs due to irreversible modifications of existing ECM proteins such as oxidation, deamidation, carbonylation, glycation, succination, and carbamylation.^32^ Because many ECM proteins, particularly collagen, have very long half-lives (on the order decades), they are particularly susceptible to buildup of these pathogenic modifications as we age.^33^ The best-studied of these are the formation of advanced glycation end-products, or AGEs. These occur via a nonenzymatic reaction between reducing sugars and proteins, lipids, or nucleic acids.^34^ AGEs modify binding domains and alter the mechanical function of ECM proteins, particularly collagens, which lead to overall aberrant ECM topography. This stiffens the tissue, reducing critical viscoelastic function, which confers age-related pathologies.^35^ Similarly, matrix metalloproteinases (MMPs) are normally responsible for ECM breakdown to maintain homeostasis and ensure proper tissue remodeling.^36^ However, MMP activity becomes elevated as we age, resulting in aberrant ECM organization via accumulation of fragmented, heavily cross-linked collagen and loss of critical ECM ligands.^37^ In the context of cancer, these changes create an environment that is permissible for cancer cell attachment and outgrowth. In particular, it has been well-documented that primary tumors modify their ECM to form a stiffer, more permissive stroma that promotes cancer cell endothelial to mesenchymal transition,^38^ endothelium interaction,^39^ and migration.^21,40^ Thus, age-related biomechanical changes that occur in the ECM throughout the body potentiate cancer permissive niches that, when cancer cells reach the organ site, are primed for metastatic outgrowth.

## 3 | THE LUNG: PHYSIOLOGY, ECM, AND AGING

The pulmonary system functions to facilitate gas exchange from the air into the circulatory system. More specifically, the alveoli within the lungs act as the site of gas exchange where environmental oxygen diffuses into red blood cells in extensive capillary beds.^41^ Upon inhalation, there is a corresponding increase in lung volume, which decreases lung pressure causing air to enter the lungs. Upon exhalation, the lungs recoil to create high pressure, which forces air out of the lungs.^42^ Successful ventilation necessitates balancing lung compliance, or the willingness of lungs to distend, and elastance, or the willingness of lungs to return to resting position.^43^ Thus, our ability to breathe relies heavily on the unique elastic recoil properties of the tissue. The interstitial ECM of the lung is largely what supports these critical biomechanics.^44^ Collagen in the interstitial ECM is chiefly responsible for tensile strength, whereas elastin aptly ensures organ elasticity.^45,46^ The roles of these ECM residents become especially evident when considering pathologies that affect collagen or elastin in the interstitial ECM.

Idiopathic pulmonary lung fibrosis is a sporadic, progressive disease that causes lung scarring (fibrosis). During the course of this pathology, the collagen matrix becomes thicker largely due to increased expression of lysyl oxidases, which function to cross-link collagen in the matrix.^47^ As the interstitium thickens, so does the diffusion barrier, which inhibits the ability of the lungs to complete gas exchange as efficiently. In the case of emphysema, this pathology is characterized by a loss of elastin and concurrent breakdown of alveolar walls.^48^ This subsequently leads to progressive loss of airway functionality and is extremely difficult to remedy due to the complex somatic process required to repair elastin^49^ Overall, alterations in the levels and ratios of these proteins cause ECM stiffness and inhibit the physiological function of the lung.^46^

**FIGURE 1:**
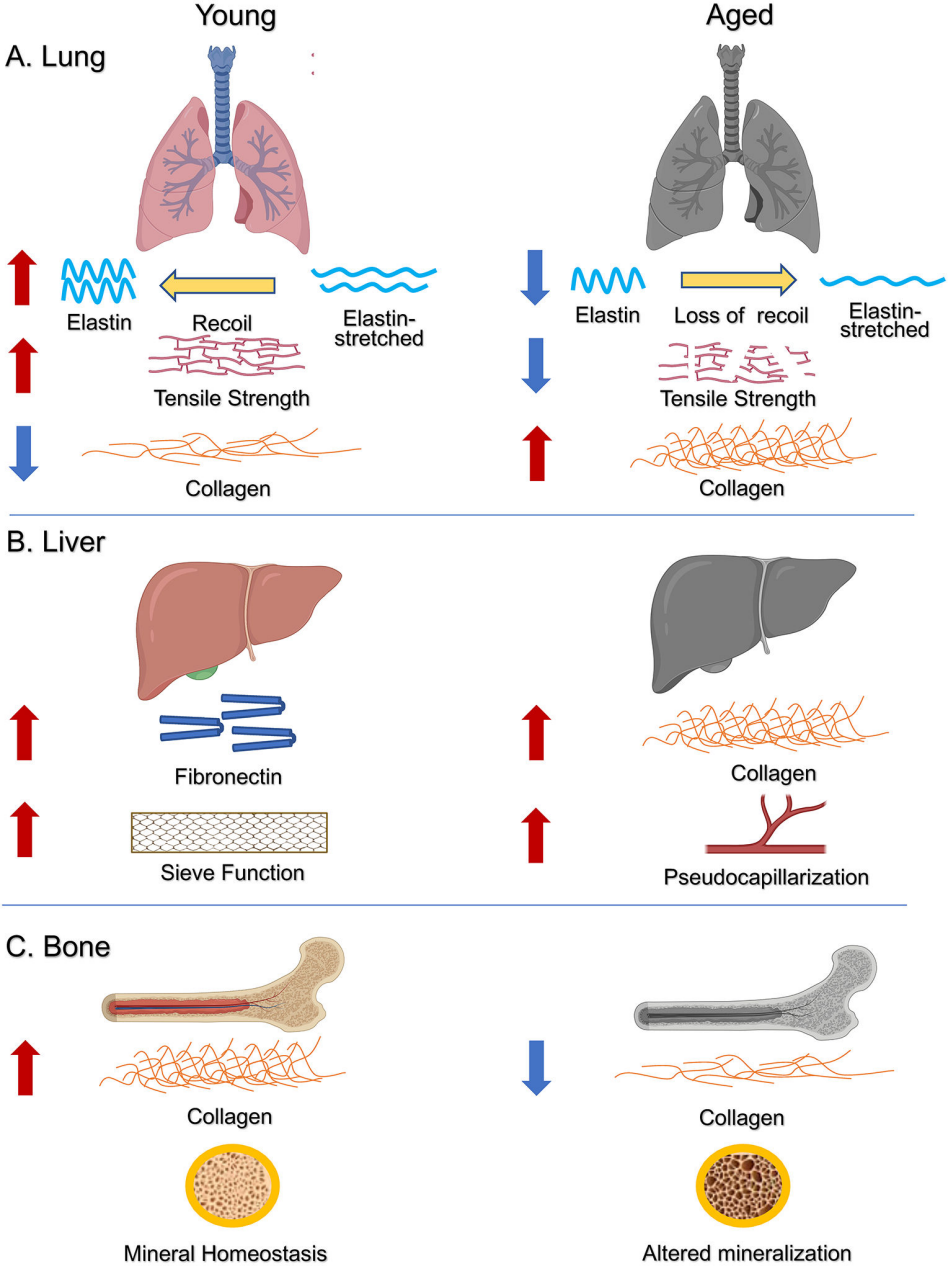
Age-dependent changes in the extracellular matrix (ECM) of the lung, liver, and bone. As we age, each of these organs incur alterations in the composition and distribution of the extracellular matrix that translate into loss of physiological function. A, Our lung relies on tissue elasticity for effective gas exchange. However, the combined effects of elastin loss and collagen accumulation with age translate into an imbalance in lung tensile strength. This decreases the elastic recoil of the lung and leads to an overall decrease in breathing capacity. B, The liver normally acts as a sieve, where fenestrated vessels effectively filter xenobiotics. As we age, the liver becomes more fibrotic, which is defined by an increase in collagen deposition. When collagen accumulates around these vessels, it causes “pseudocapillarization.” This effectively seals the vessels, which significantly diminishes the liver filtering function. C, The bones rely on delicate matrix homeostasis that balances both mineral and organic constituents to ensure skeletal strength. As we age, this homeostasis is disrupted. The imbalance resulting from loss of collagen and increased mineral content leads to decreased bone strength. Significantly, these age-dependent changes are phenocopied in numerous age-related pathologies including cancer. In realizing that aging, disease, and cancer all confer similar ECM aberrancies, we can begin to understand how these work in tandem to promote cancer progression and decrease survival in older patients. Figure created with Biorender.com

In an unbiased mass-spectrometry screen, 32 matrisome proteins were found to be significantly altered in the lung tissue proteome of aged compared to young mice. The exact proteins and data can be explored in depth here https://theislab.github.io/LungAgingAtlas.^50^ This work showed that intrinsic aging alone is sufficient to alter the ECM profile of the lung, independent of environmental or genetic impact. Interestingly, the aging lung shares many similarities with pathogenic lungs due to age-dependent alterations in the ECM. In fact, ECM degradation has been defined as a hallmark of the aging lung.^51,52^ As we age, elastin in and around the respiratory bronchiole begins to degenerate, and loses its recoil pressure.^53^ This, in turn, decreases the ratio of surface area to lung volume in an approximately linear manner throughout life, resulting in a 25–30% decrease in airway capacity at age 90.^54^ This phenomenon has been coined “senile emphysema,” where the aged lung has the properties of emphysemic lungs due to ECM degradation without concurrent destruction of the alveolar wall that marks “true” emphysema.^55^ When comparing native and decellularized lung between young and aged mice, aged mice tend to have less elastin, more collagen, and overall decreased variety of ECM components than young mice.^56^ This is consistent with what is seen in patients with idiopathic pulmonary fibrosis and chronic obstructive pulmonary disease, causing a relative decrease of tissue elastance and resistance with age. This makes aged lungs more susceptible to such pathologies, while also inhibiting the proper biomechanics of breathing^57^ (Figure 1A).

## 4 | THE LIVER: PHYSIOLOGY, ECM, AND AGING

The liver is a complex and multifaceted organ that exerts numerous functions to support bodily health. Its primary function is to filter the blood and rid the body of xenobiotics, but it also creates and secretes bile and acts as the epicenter for metabolism.^58^ The interstitial ECM of the liver is primarily made up of collagen and fibronectin.^59^ The hepatic stellate cells are the primary effector cells for producing and depositing ECM in the liver and reside in the subendothelial space beneath the sinusoidal cells.^60,61^ They are especially relevant in the wound-healing response where, upon activation, they reduce their fat storage and produce collagen.^62^ However, whether these cells generate ECM during normal homeostasis is still unclear as other cell types such as fibrocytes, periportal fibroblasts, and myofibroblasts have been implicated in ECM depositon.^63^ The liver is a unique organ in that it is capable of fully regenerating, largely due to rapid hepatocyte mitosis, but also due to increased deposition of lost ECM by these players.^59^ The ECM makes up a very small portion of liver area, less than 5%, but because of where the ECM is concentrated, minute alterations have a significant impact on overall organ physiology. Notably, the ECM located in the space of Disse (the space between the liver epithelium and the sinusoidal endothelium) is extremely fragile. If there is an increase in ECM fibrillar protein deposition, it can “seal up” or “capillarize” the sinusoids, effectively removing the sieve properties of the vessels.^64,65^ This in turn impedes the ability of the liver to filter blood as effectively, and results in increased oxidative stress.^66^ This phenomenon is also common in patients with cirrhosis, chronic hepatitis, and alcoholic liver disease.^67,68^

Additionally, the ECM of the liver is normally relatively elastic, with a baseline stiffness of 2 kPa. However, when liver becomes fibrotic such as in chronic liver disease, stiffness can increase to up to 8 kPa.^69^ This causes aberrancies in both the structure and composition of the hepatic ECM, which inhibits its ability to regulate the growth of both hepatocyte and nonhepatocyte liver cells.^70^ During the normal aging process, the liver often phenocopies these pathologies.^66^ Although young livers tend to have minimal ECM in the space of Disse, as we age, so-called pseudocapillarization occurs upon increased deposition of collagen in this space. This leads to reduced fenestration and thickening of the endothelium.^71^ Additionally, it has been shown that aging causes an increased baseline activation state of hepatic stellate cells, which in turn increases collagen deposition and increases tissue stiffness.^72^ This also leads to an overall pro-inflammatory state in the tissue, which creates a vicious cycle of further stellate cell activation and fibrosis (Figure 1B). Thus, these age-dependent changes in the ECM inhibit the normal function of the liver and cause symptoms that mimic liver-related pathologies.

## 5 | THE BONES: PHYSIOLOGY, ECM, AND AGING

Our skeletal system functions to provide structural support to the body, protect our organs, and potentiate motion. On a cellular level, the bones maintain mineral homeostasis and provide the environment for hematopoiesis in the marrow. Bone and bone mineral matrix are produced by osteoblasts and regulated by osteoclasts, which break down bone.^73^ The bone ECM is unique in that it is highly mineralized (comprising 50–70% of its total volume) as opposed to organic.^74^ The major mineral content is hydroxyapatite, which forms tiny crystals within the matrix.^75^ This crystalline component provides the mechanical rigidity and load-bearing strength required for skeletal function, whereas fibrillar proteins, mostly collagen I, provide flexibility. Although collagen does not provide strength to the bones per se, it is critical for impact resistance as well as regulating tissue remodeling.^76,77^

The critical role of the bone ECM has been extensively studied in the age-associated disease, osteoporosis. In addition to loss of bone density, osteoporosis is marked by a hypermineralized ECM that reduces bones’ ability to withstand impact and leaves them more prone to micro- and macrofracture.^78,79^ As we age, numerous changes occur in the bone ECM that inhibit physiological function. Accumulation of AGEs and other posttranslational modifications of bone ECM fibular proteins has been shown to inhibit the regeneration of bone, leading to brittleness and increased propensity for subsequent pathologies.^80^ It has also been shown that there is an age-dependent loss of collagen in the bone ECM, which disrupts the organic balance of the matrix rendering older bones less “tough”^81^ (Figure 1C). Thus, as with the liver and lung a theme emerges: increased stiffness is the enemy of physiological function.

## 6 | THE ECM AND CANCER: INTERPLAY AND SIMILARITIES TO THE AGING PHENOTYPE

Just as in aging, the ECM alterations we see in malignant tissues confer loss of matrix homeostasis and increased organ stiffness. In primary tumors, cancer cells reorganize their surrounding matrix almost immediately to begin forming a cancer-permissive substrate.^82,83^ In effect, they hijack dynamic reciprocity in order to secure their own survival to the detriment of organ health. The resulting stiffening and degradation of the ECM drives cancer proliferation, survival, invasion, and metastasis.^84^

Approximately 30% of patients with lung adenocarcinoma have a loss of function mutation in LKB1—the negative regulator of lysyl oxidase. Without inhibition, an abundance of lysyl oxidase allows for increased collagen deposition in the surrounding ECM, which stiffens the lung and confers increased proliferation and invasiveness of adenocarcinoma cells. In patients, high levels of lysyl oxidases are correlated with decreased survival.^85^ Thus, we see that lysyl oxidase mediates cancer-associated stiffness in a similar manner to that observed in idiopathic pulmonary lung fibrosis as well as intrinsic aging. In addition to proliferation, this stiffer lung ECM allows for increased expression of PDL1 on lung adenocarcinoma cells, which allows them to escape immunosurveillance and ensure survival.^86^ Approximately 80–90% of all hepatocellular carcinomas develop in patients with fibrotic or cirrhotic livers, which renders these the strongest predisposing factors for liver malignancy.^87^ We know that this can occur both through various pathologies (alcoholic liver disease, chronic hepatitis, etc) and through the natural aging process. In fact, it has been shown that matrix stiffness can act as an independent initiator of epithelial-to-mesenchymal transition in hepatocellular carcinoma, and those patients with stiffer livers tend to have higher incidence of cancer recurrence.^88^ Finally, the ECM of osteosarcoma patients is extremely similar to that observed in osteoporosis patients. As we age, the rate of bone turnover decreases significantly, which increases the brittleness of our bones and alters the matrix mineral structure. Although slower bone turnover is known to be protective against bone metastasis,^89^ this effect is paradoxically diminished in the aged system. The delicate balance of osteoblast/osteoclast bone homeostasis is normally regulated by expression of RANKL and RANK, respectively.^90^ However, osteosarcoma patients often present with upregulated RANKL, which functions to stimulate osteoclasts and accelerate bone and matrix degradation. This allows for the release of latent growth factors embedded in the matrix, which stimulates cancer cell proliferation.^90,91^ Subsequently, activation of osteoclasts stimulates osteoblasts to form new bone and matrix, increasing the likelihood of metastatic outgrowth.^92^

Taken together, it is clear that cancer cells modulate ECM stiffness and composition to form an ideal environment for growth and invasion. Interestingly, the prerequisite for a tumor-permissive ECM is strikingly similar to what is observed to be a consequence of intrinsic aging. Although cancer is often called “a wound that will not heal,” in part because of its similarities to scar tissue and other fibrotic pathologies,^93^ we also must consider cancer as a disease of aging in the same breath. Thus aging, pathology, and cancer all confer similar ECM aberrancies, albeit to varying extent, that promote disease and inhibit physiological function, leading to a decline in bodily health.

## 7 | THE AGED ECM AS A PREMETASTATIC NICHE

The premetastatic niche can be thought of as tilling the “soil” to prepare it for successful “seed” implantation.^94^ Thus, in order for successful metastatic outgrowth, the site must be appropriately primed. It is known that formation of the premetastatic niche is largely dictated by significant alterations in the deposition and accumulation of ECM.^95^ As outlined in this review, it is clear that age-related pathologies that are characterized by altered ECMs show similar phenotypes to what is seen in malignancy. Accordingly, alterations in the ECM of future sites of metastasis have been demonstrated in numerous cancer models.^96^ Decellularized matrix from the liver and lung of tumor-bearing mice significantly increases tumor cell adhesion compared to ECM from healthy mice.^97^ In the bones, upregulation of lysyl oxidase occurs in tandem with primary tumor formation, which increases bone stiffness and allows a more growth-permissive environment for cells that eventually intravasate.^98,99^

It is worth noting that age-associated alterations in the ECM also potentiate other key players to form a complete “ecosystem” in the premetastatic niche. First, the immune system undergoes significant age-related changes that impact cancer progression. Immunosenescence is defined as the gradual, age-related decline in immune cell function, affecting the adaptive immune response more dramatically than the innate. Immunosenescence is highly detrimental as it decreases the ability to effectively administer an immune response.^100^ This is crucial for cancer progression, as this allows for metastatic outgrowth at distant sites to proceed unabated by the immune system.^101^ Interestingly, age-dependent breakdown of the ECM has been hailed as a hallmark of immunosenescence, as this alters immune infiltrate dynamics by hindering their ability to mobilize and effectively target both pathogens and cancer cells.^102,103^

As we age, a greater proportion of all of our cells become senescent.^104^ As evidenced by the principal of dynamic reciprocity, there is similarly significant cross talk between senescent cells and their ECM. As cells enter senescence, their secretome profile changes considerably to exacerbate fibrosis of the ECM.^105^ Conversely, the ECM is able to regulate cellular senescence via ligand/receptor interactions.^106^ Senescent cells often present a roadblock in cancer therapy, as they are frequently resistant and promote tumor metastasis and relapse.^107^

All cells in our body must interpret signals from the ECM via mechanotransduction in order to properly regulate function.^108^ We have discussed that the ECM becomes stiffer with age, which alters the organ-specific compliance necessary for healthy function. These changes in biomechanics are interpreted by cells and result in aberrant feed-forward loops that can accelerate disease progression, including cancer. Notably, YAP and TAZ are transcriptional coactivators that are chiefly responsible for converting external mechanical cues into genetic alterations.^109^ YAP and TAZ are overexpressed in numerous cancer types, and their transcriptional program increases key malignant phenotypes including cell proliferation and survival.^110^ In the context of age-induced stiffening, this baseline stress lowers the threshold for YAP/TAZ activation, causing heightened transcriptional activity, which can lead to acceleration of cancer.^111^

Last, the ECM plays a critical role in exerting selective pressure on tumor cell populations. Primary tumors exhibit numerous different populations of cells with distinct molecular signatures as a consequence of selective pressures over the course of disease.^112^ As tumors grow, corresponding region-specific alterations in oxygen availability, ECM topography, and immune response influence cancer cell survival.^113^ In order to survive in an increasingly hostile environment, cancer cells adapt to form a mosaic of populations within a single tumor.^114,115^ Importantly, these external pressures and subsequent genetic changes are vital for the ability of cancer cells to gain metastatic competency.^116^ Although little work has been done to definitively show that the *aged* ECM contributes to this transition, many age-related ECM changes are known to accelerate the evolution of aggressive tumor populations. For instance, cancer cell migratory capacity is known to operate in a biphasic manner based on ECM stiffness.^117^ It occurs such that increasing density increases cell invasion to a point, after which fiber pore size becomes too small for cell motion.^118^ Thus, age-related changes in ECM stiffness may lead to increased metastatic potential by altering the baseline matrix dynamics to “push” cancer cells into a more invasive state.

**FIGURE 2:**
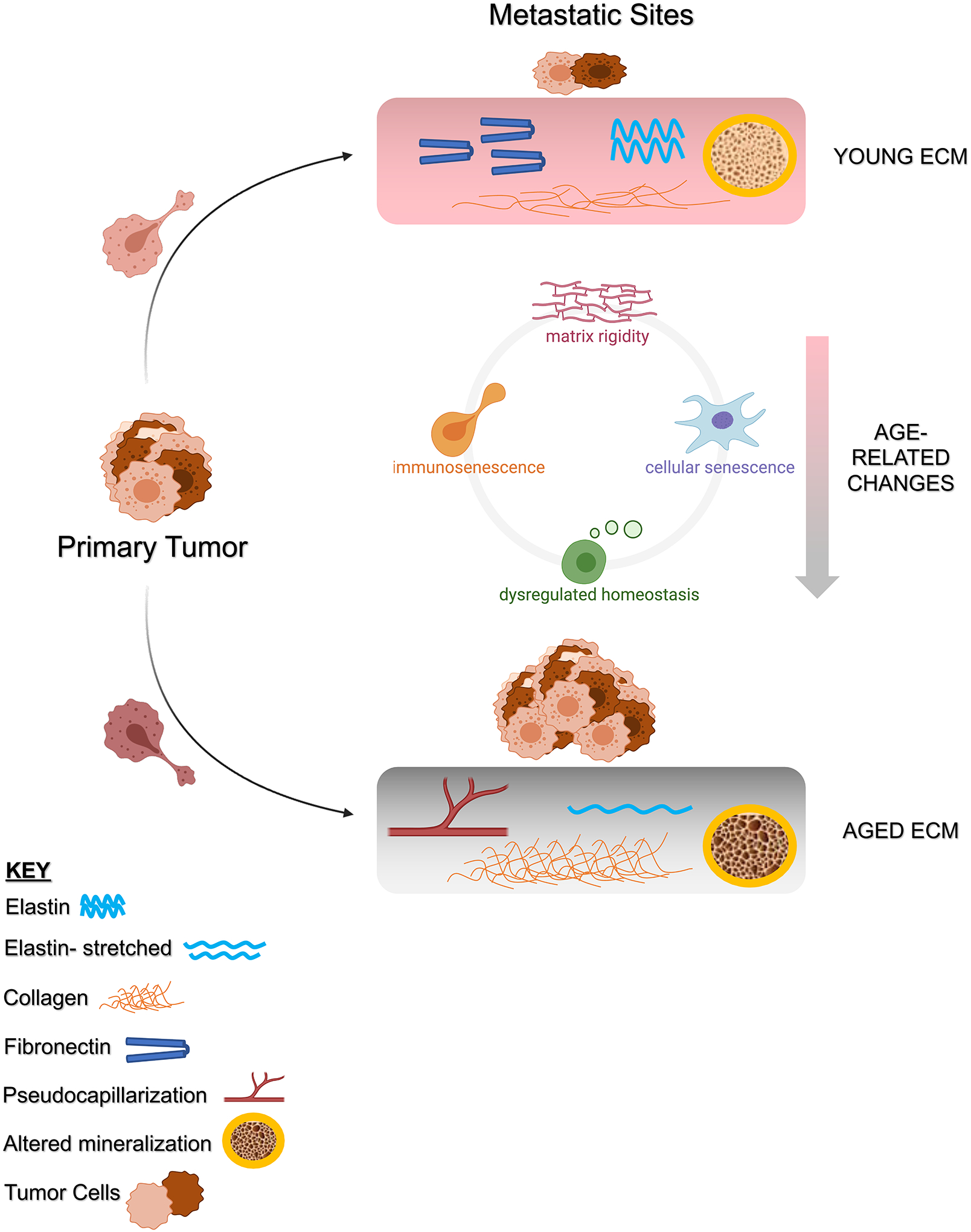
Age-related changes in the extracellular matrix (ECM) prime future sites of metastasis. When cancer cell “seeds” escape the primary tumor site, they are more likely to grow at a distant “soil” site they deem favorable. Normally, our organs have pliable ECM, with a balance of fibronectin, collagen, elastin, and overall homeostasis. Essentially when we are young, our organ ECMs support appropriate biomechanical function and overall health. Such an environment is unfavorable to cancer cell growth, and thus metastatic outgrowth is inhibited. However, aging induces numerous chances to the ECM in our organ systems. Overall, this includes increased matrix rigidity/stiffness and dysregulated homeostasis. This leads to an imbalance in ECM constituents, most commonly by excess collagen deposition. The resulting stiffness manifests in loss of elasticity (lung), pseudocapillarization (liver), and mineral imbalance (bones). These changes also exacerbate immunosenescence and cellular senescence, which help form a more complete premetastatic niche. In all, the aging process forms fertile “soil” that is favorable to cancer cell survival and growth. More studies are needed to explore the direct link between intrinsic aging and metastasis. Specifically, work should focus on ways to target the aged ECM of the lung, liver, bone, and others, to disrupt the metastatic cascade with the goal of improving outcomes for the aged patient population. Figure created with Biorender.com

Together, age-dependent ECM changes both directly influence the formation of cancer-permissive niches as well as influence other cell populations further exacerbating cancer cell metastasis at distant sites. Overall, the aging process itself seems to play a significant role in forming the premetastatic niche and, consequently, in driving cancer progression and metastatic outgrowth (Figure 2). However, there is a noticeable absence of research directly investigating the link between age-related changes in the ECM and how these prime future sites of metastasis.

## 8 | CONCLUDING REMARKS

Aging is a poor prognostic factor for most types of cancer, and older patients tend to present with more aggressive and advance-stage disease. As the aging population increases both in the United States and around the world, understanding the implications of intrinsic aging on cancer progression is more critical now than ever. It has become clear since Paget first posited “seed and soil” over 130 years ago that the ECM is indispensable for successful metastatic outgrowth. Although hematopoietic physiology plays a role in determining the common sites of metastasis, it is the alterations in the ECM of these organ sites that potentiate outgrowth and lead to advance-stage disease.

We understand that the ECM of the lung, liver, and bone changes as we age. We also understand that these changes are consistent with what we know to be tumor-permissive phenotypes. Thus, it is reasonable to suggest that intrinsic aging is sufficient to prime these organ sites for metastatic outgrowth. This would help explain why older cancer patients progress so quickly and present at later stages more frequently: if the ECMs of their organs are already tumor permissive, minimal remodeling is required for metastatic outgrowth. However, little research has been done to definitively prove that intrinsic aging at the site of metastasis is sufficient for cancer progression and outgrowth. Some recent work has been done to try to target the metastatic ECM. For example, nanobody libraries against metastasis-associated ECM proteins have been used to visualize small metastases, but could potentially be used to target the ECM, or disrupt ECM/cancer cell interaction.^119^ However, the vast majority of ECM therapies have been directed at various fibrotic pathologies and the sites of primary, but not distal, tumors. Few, if any, of these have been tested in the context of metastasis or intrinsic aging. Thus, there is a critical need to expand our knowledge in this area. Future investigations should focus on how intrinsic aging alters the ECMs of the lung, liver, bone, and others, and how age-associated changes impact subsequent metastatic progression to these sites. Further, we will need to determine whether targeting either ECM components or cell/ECM interactions in the aged context might alleviate metastatic burden. It would also be prudent for future studies to address whether alleviating age-related ECM degradation at metastatic sites prior to tumor challenge is sufficient to reduce metastasis and slow tumor progression. It will also be important to understand which cancer types do not follow this paradigm of increasing age driving metastasis, and why. Apart from basic science research, it is critical that preclinical studies take age into account to better understand both the application and function of potential therapies. This will also help us understand the biomechanistic underpinnings of the aging process and inform our understanding of how it influences cancer progression. Together, by understanding the intersection of aging, metastasis, and the ECM, we will be well-equipped to rise to the challenge of improving the lives and outcomes of older cancer patients.
